# A Phase I Trial of Dasatinib and Osimertinib in TKI Naïve Patients With Advanced EGFR-Mutant Non-Small-Cell Lung Cancer

**DOI:** 10.3389/fonc.2021.728155

**Published:** 2021-09-08

**Authors:** Chul Kim, Stephen V. Liu, Jennifer Crawford, Tisdrey Torres, Vincent Chen, Jillian Thompson, Ming Tan, Giuseppe Esposito, Deepa S. Subramaniam, Giuseppe Giaccone

**Affiliations:** ^1^Department of Oncology, Lombardi Comprehensive Cancer Center, Georgetown University, Washington, DC, United States; ^2^Department of Biostatistics, Bioinformatics & Biomathematics, Georgetown University, Washington, DC, United States; ^3^Department of Radiology, Georgetown University Hospital, Washington, DC, United States; ^4^AstraZeneca Plc., Gaithersburg, MD, United States; ^5^Weill-Cornell Medicine, New York, NY, United States

**Keywords:** EGFR, Src, osimertinib, dasatinib, lung cancer

## Abstract

**Background:**

Osimertinib is an effective first-line therapy option for *EGFR*-mutant NSCLC, but virtually all patients develop resistance. CRIPTO, through Src activation, has been implicated in resistance to EGFR tyrosine kinase inhibitor (EGFR-TKI) therapy. Dasatinib, a Src inhibitor, has shown preclinical synergy with EGFR-TKI therapy.

**Method:**

This is a single-arm phase I/II trial of osimertinib and dasatinib in TKI-naïve advanced *EGFR*-mutant NSCLC (NCT02954523). A 3 + 3 design was used in the phase I to establish the recommended phase II dose (RP2D). Osimertinib 80 mg QD was combined with dasatinib 70 mg BID (DL2), 50 mg BID (DL1), 70 mg QD (DL-1), and 50 mg QD (DL-2).

**Results:**

Ten patients (DL2: 3, DL1: 6, DL -1: 1) were enrolled. 3 (50%) of 6 patients at DL1 experienced a DLT (grade 3 headaches/body pain, neutropenia, rash, one each). Common treatment-related adverse events included pleural effusion (n=10), diarrhea (n=8), rash (n=7), transaminitis (n=7), thrombocytopenia (n=7), and neutropenia (n=7). While the MTD was not determined by protocol-defined DLT criteria, DL-2 was chosen as the RP2D, considering overall tolerability. Nine (90%) patients had a PR, including 1 unconfirmed PR. Median PFS was 19.4 months and median OS 36.1 months. The trial was closed to accrual prematurely due to slow accrual after the approval of osimertinib as first-line therapy.

**Conclusions:**

The combination of dasatinib and osimertinib demonstrated anticancer activity. The treatment was limited by chronic toxicities mainly attributed to dasatinib. To improve the safety and tolerability of Src and EGFR co-inhibition, Src inhibitors with a more favorable safety profile should be utilized in future studies.

**Clinical Trial Registration:**

https://clinicaltrials.gov/ct2/show/NCT02954523

## Introduction

Lung adenocarcinoma, the most common subtype of lung cancer, frequently harbors oncogenic driver mutations in the epidermal growth factor receptor (EGFR). EGFR tyrosine kinase inhibitor (TKI) therapy has emerged as the standard first-line treatment option for *EGFR*-mutant non-small cell lung cancer (NSCLC) (1). Osimertinib, a third-generation EGFR-TKI, has been shown to improve both progression-free survival (PFS) and overall survival (OS) compared with first-generation EGFR-TKIs in the randomized phase III trial FLAURA (2, 3). In FLAURA, the objective response rate (ORR) with osimertinib was 80% with median duration of response of 17.2 months, and the median PFS was 18.9 months. Although the majority of patients with lung adenocarcinoma harboring sensitizing *EGFR* mutations respond to EGFR-TKIs including osimertinib, efficacy is limited by the universal development of acquired resistance. Moreover, a small fraction of patients experience intrinsic resistance to EGFR-TKIs, defined by progressive disease or non-durable stable disease.

We previously showed that tumor overexpression of CRIPTO, a membrane-bound and secreted protein of the EGF-CFC family that is commonly referred to as TDGF1, results in intrinsic resistance to early-generation EGFR-TKIs (4, 5). CRIPTO desensitizes *EGFR*-mutant NSCLC tumor cells to EGFR-TKI treatment through CRIPTO-mediated Src activation, and inhibition of Src resensitizes CRIPTO-expressing cells to EGFR-TKI treatment. EGFR-TKI therapy combined with a Src inhibitor produces a synergistic antitumor effect against CRIPTO positive/*EGFR*-mutant xenograft tumor models. Furthermore, expression of CRIPTO was significantly higher in the *EGFR*-mutant NSCLC tumors from EGFR-TKI non-responders than in those from responders. These data indicate that CRIPTO through Src activation is an important contributor to the resistance to EGFR-TKIs.

We hypothesized that osimertinib, in combination with the Src inhibitor dasatinib would act synergistically to overcome resistance to EGFR-TKIs. Here we report the final results of a phase I study of osimertinib and dasatinib in TKI-naïve patients with *EGFR*-mutated NSCLC.

## Materials and Methods

### Study Design and Objectives

This was a single-center open-label, single arm phase I/II trial of osimertinib and dasatinib (NCT02954523). The standard 3 + 3 design was used for dose escalation (6). The primary objectives for the phase I portion were to evaluate the safety and tolerability of the combination therapy and to determine the maximum tolerated dose (MTD) and recommended phase II dose (RP2D) of osimertinib when given in combination with dasatinib in patients with TKI-naïve advanced *EGFR*-mutant NSCLC. The primary objective for the phase II portion was to determine the rate of non-response to the combination of osimertinib and dasatinib in patients stratified by CRIPTO expression in their tumor. Secondary objectives included assessment of plasma pharmacokinetics and clinical outcomes including PFS, OS, and duration of response (DOR). Exploratory objectives included assessment of the role of fluorodeoxyglucose (FDG)-positron emission tomography (PET) obtained on day 28 in predicting response to study treatment and evaluation of plasma CRIPTO level and its association with treatment outcomes. The study conformed to the Declaration of Helsinki and Good Clinical Practice guidelines. The study was approved by the Georgetown Institutional Review Board and the United States Food and Drug Administration (FDA).

### Study Treatment

The dose of osimertinib was 80 mg once a day across all dose levels. At dose level 1 (starting dose level), patients received osimertinib 80 mg once daily plus dasatinib 50 mg twice daily. At dose level 2, the dose of dasatinib was 70 mg twice daily. There were two dose levels below the starting dose level if dose reductions were required (dose level -1: osimertinib 80 mg once daily and dasatinib 70 mg once daily, dose level -2: osimertinib 80 mg once daily and dasatinib 50 mg once daily). Treatment was administered continuously in 28-day cycles. For treatment-related adverse events, dose reductions were allowed with the aim of maintaining a full dose of osimertinib and reducing primarily dasatinib unless the toxicity was clearly associated with osimertinib. Patients were allowed to stay on treatment beyond progression if deemed in the patient’s best interest.

### Study Patients

Patients with advanced NSCLC harboring sensitizing *EGFR* mutations (deletion in exon 19, L858R in exon 21, G719X, and L861Q) were eligible. No prior treatment with an EGFR tyrosine kinase inhibitor was allowed. Patients were eligible if they had an Eastern Cooperative Oncology Group (ECOG) performance status of 0–2, adequate organ/bone marrow function, and measurable disease. Patients with untreated symptomatic brain metastases were eligible if the symptoms did not warrant urgent surgery or radiation, and treatment with steroids was not necessary. Pleural or pericardial effusions of any grade were not allowed, but patients whose pleural/pericardial effusions were resolved at the time of study entry were eligible.

### Pharmacokinetic Studies

Blood samples were collected at the following time points: on cycle 1 day 1, predose, and 2-, 4-, and 6-hour postdose; on cycle 2 day 1, predose and 4-hour postdose; on day 1 of subsequent cycles, predose. Descriptive pharmacokinetics utilizing visual inspection was used to determine maximum concentration (Cmax) and time of Cmax (Tmax) of osimertinib and its metabolite, AZ13575104, following the administration of osimertinib and dasatinib on cycle 1 day 1. Protein precipitation followed by liquid chromatography with tandem mass spectrometry (LC-MS/MS) was used to determine the concentrations of osimertinib and its metabolite AZ13575104 (7). Since the method used for sample analysis had previously been validated in EDTA anticoagulant only, and the study samples were collected using lithium heparin anticoagulant, a qualification run confirmed that osimertinib and AZ13575104 in plasma, containing lithium heparin anticoagulant, can be quantified in study samples using the validated calibration curve and QC samples in EDTA anticoagulant. Calibration standard data, quality control (QC) sample data, incurred sample reanalysis data and chromatograms indicated that the method performed acceptably during the sample analysis. In co-administered drug assessment, dasatinib did not affect the quantification of osimertinib or AZ13575104. Dasatinib pharmacokinetics were not assessed.

### Evaluation of Levels of Serum CRIPTO

Quantification of secreted CRIPTO levels in serum was performed using the ELISA kit (Catalog numbers DY145 & DY008) from R&D Systems (Minneapolis, Minnesota, USA). Blood samples were collected every cycle, centrifuged at 1400 RPM for 10 mins, and serum was aliquoted and frozen at −80°C until analysis. Protein standards provided by the kit were diluted and read using the GloMax-Multi Detection System (Promega, Madison, Wisconsin, USA) at 450 nm and 560 nm. Absorbance values at 450 nm were subtracted by values at 560 nm to generate a protein standards curve. All ELISA values from samples were determined by absorbance detection at 450 nm and 560 nm, subsequent subtraction of 450 nm values with 560 nm values, and subsequent plotting on the protein standards curve.

### Safety and Response Evaluation

Patients were evaluated every two weeks during cycle 1. Once treatment tolerance was established, patients were seen on day 1 of each cycle or more frequently if medically indicated. Prior to each cycle, history and physical exam, laboratory evaluation including complete blood count (CBC), comprehensive metabolic panel (CMP), and electrocardiogram (EKG) were performed. An echocardiogram or a multigated acquisition (MUGA) scan was done at baseline and as clinically indicated thereafter. The dose limiting toxicity (DLT) period consisted of one cycle (28 days). DLT was defined as adverse events occurring during the first cycle of therapy and related to the study treatment while fulfilling one of the following criteria as per Common Terminology Criteria for Adverse Events (CTCAE) version 4.03: 1) any grade 3 or 4 toxicity except for grade 3 diarrhea, nausea, or vomiting controlled with supportive therapy; 2) persistent (>21 days) non-hematologic grade 2 adverse events despite optimal medical management; and 3) treatment delay > 21 days. Determination of the RP2D was based on the maximum tolerated dose (MTD), tolerability, and toxicities beyond cycle 1. Tumor assessment was performed every 2 cycles. Response to treatment was evaluated using the international criteria proposed by the Response Evaluation Criteria in Solid Tumors (RECIST) version 1.1 (8).

### Statistical Considerations

Descriptive statistics were used to summarize treatment outcome measures. The safety profile of the study treatment was assessed through summaries of dose-limiting toxicities (DLTs) and treatment-related AEs (TRAEs). PFS and OS were estimated and plotted by the Kaplan–Meier method. For the phase II portion, a total of 28 patients were to be enrolled using a two-stage group sequential design (9), testing the null hypothesis that the proportion of patients who progress or have stable disease lasting 4 months or less is at least 30% against the alternative hypothesis that the true proportion of patients who progress or have stable disease ≤ 4 months is 10% with 85% power at a significance level of 5%. The data cutoff date was December 11, 2020. GraphPad Prism 9.0.0 and R (survfit) was used for data analysis.

## Results

### Patient Characteristics

A total of 10 patients were enrolled in the trial (**Table 1**). Nine (90%) patients were female. The median age at enrollment was 70.5 years (range: 48-83). Eight (80%) patients had adenocarcinoma, 1 (10%) had adenosquamous carcinoma, and 1 (10%) had undifferentiated carcinoma. Six (60%) had *EGFR* exon 19 deletions and 4 (40%) had the *EGFR* L858R point mutation. Four (40%) patients had brain metastases at baseline.

**Table 1:** Patient characteristics.

Median age		70.5 (range: 48-83)
**Sex**	Female	9 (90%)
	Male	1 (10%)
**Race**	White	5 (50%)
	Asian	5 (50%)
**Smoking status**	Never	7 (70%)
	Former	3 (30%)
**Performance status**	0	5 (50%)
	1	5 (50%)
**Histology**	Adenocarcinoma	8 (80%)
	Adenosquamous	1 (10%)
	Undifferentiated carcinoma	1 (10%)
**EGFR mutation**	Exon 19 deletion	6 (60%)
	L858R	4 (40%)
**Brain metastases at baseline**		4 (40%)

### Safety and Tolerability of Osimertinib and Dasatinib

Three patients were initially enrolled at dose level 1 and did not experience any DLTs. Subsequently, three patients were enrolled at dose level 2 and no DLTs were observed. However, due to the occurrence of multiple chronic toxicities beyond cycle 1 including pleural effusion, fatigue, and nausea, frequent dose reductions of dasatinib were necessary (**Supplemental Tables 1, 2**) at dose level 1 and 2, Therefore, it was decided to expand dose level 1 instead of dose level 2. In the three additional patients who entered dose level 1, three DLTs were observed (grade 3 headache and myalgia, grade 3 neutropenia, and grade 3 rash). One patient was enrolled at dose level -1 (osimertinib 80 mg once daily and dasatinib 70 mg once daily) and did not have a DLT. No dose reductions were needed for osimertinib. We evaluated how long patients were able to stay on each dose level of dasatinib during their treatment. Average duration of treatment with dasatinib (excluding the days off dasatinib due to dose interruption) was 50, 65, 35, and 535 days on dose level 2, dose level 1, dose level -1, and dose level -2, respectively (**Supplemental Table 1**). The trial was closed to further enrollment, due to slow accrual after approval of osimertinib in the first-line setting.

Common TRAEs included pleural effusion (n=10), diarrhea (n=8), rash (n=7), thrombocytopenia (n=7), neutropenia (n=7), AST elevation (n=7), ALT elevation (n=7), nausea (n=4), QTc prolongation (n=4), anorexia (n=4), paronychia (n=4), fatigue (n=4), and anemia (n=4) (**Table 2**). Most TRAEs were grade 1 or 2 (92%). Pleural effusion is a known side effect of dasatinib; 5 patients had grade 1 pleural effusion (asymptomatic or intervention not indicated), 4 had grade 2 pleural effusion (symptomatic or intervention indicated), and 1 patient had grade 3 pleural effusion (symptomatic with respiratory distress and hypoxia). Four patients underwent thoracentesis and 2 patients required indwelling catheter placement. No grade 4 or 5 TRAEs were observed.

**Table 2:** Treatment-related adverse events experienced in more than one patient* (n=10).

	Grade 1	Grade 2	Grade 3	Total
**Hematologic**				
Thrombocytopenia	6	1		7
Neutropenia	2	2	3	7
Anemia		4		4
Leukopenia	3			3
**Non-hematologic**				
Pleural effusion	5	4	1	10
Diarrhea	4	4		8
Rash	6		1	7
AST elevation	7			7
ALT elevation	7			7
Nausea		4		4
QTc prolongation		4		4
Anorexia	1	3		4
Paronychia	1	3		4
Fatigue	1	2	1	4
Dyspnea		2	1	3
Fever	1	2		3
Mucositis	3			3
Creatinine increase		3		3
Weight loss	2	1		3
Facial edema	3			3
Palpitation	3			3
Dysgeusia	2			2
Nail discoloration	2			2
Cough	1	1		2
Alopecia	2			2
Headaches	1		1	2
Hypoxia		1	1	2
Edema of lower extremity	1	1		2
Ejection fraction decrease	1		1	2
Myalgia	1		1	2
Pneumonitis			1	1

*Pneumonitis was included as a TRAE of special interest though it was experienced in only one patient.

### Efficacy of Osimertinib and Dasatinib

**Figure 1:**
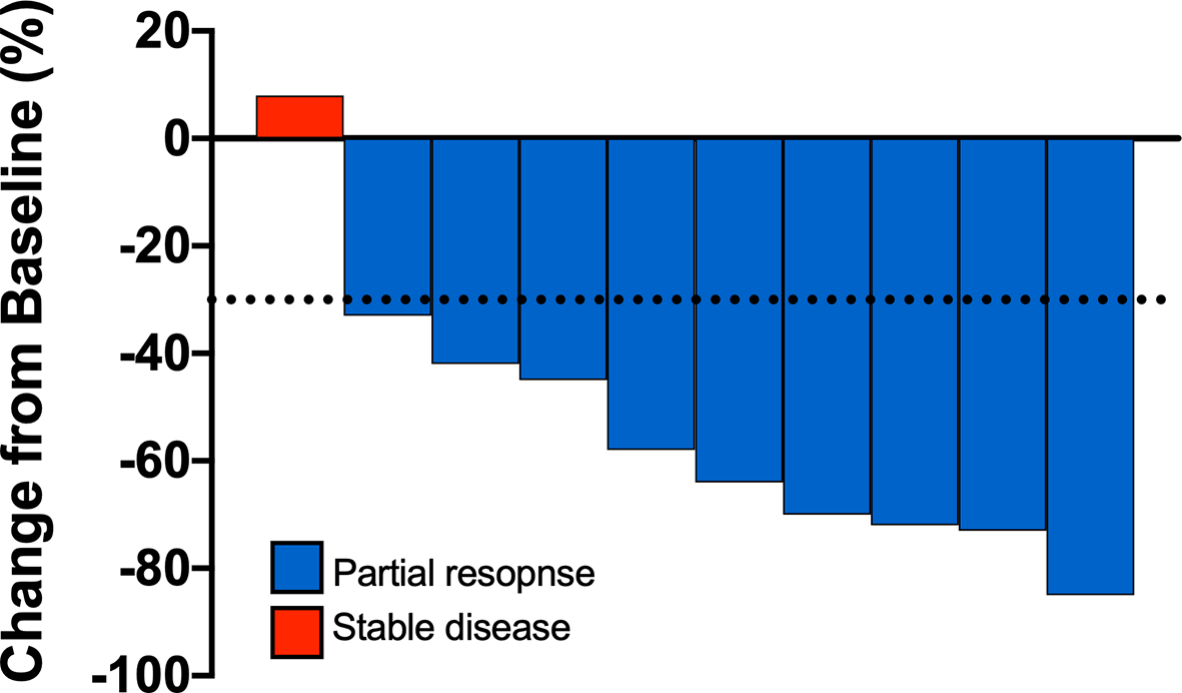
Waterfall plots are shown summarizing the best percentage change in target lesions. Each bar represents a patient.

**Figure 2:**
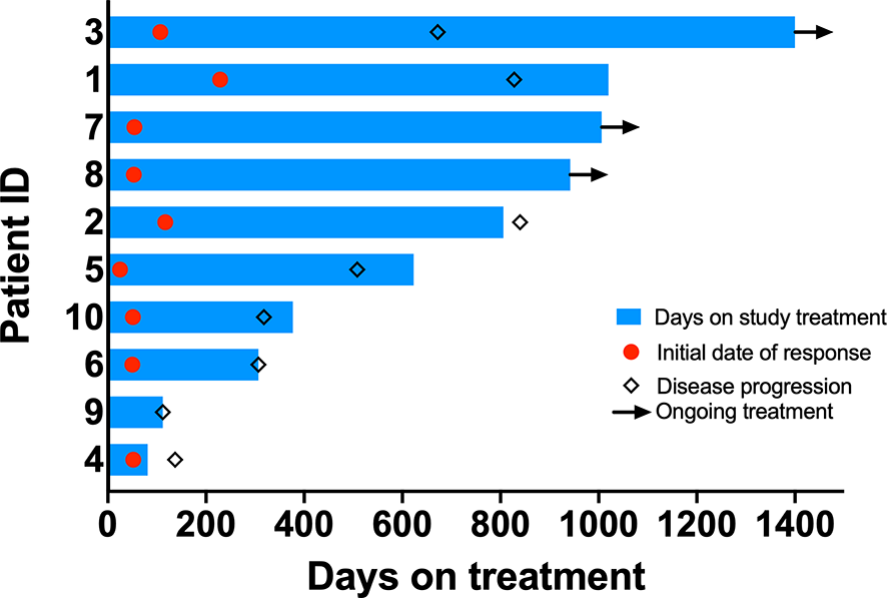
Swimmer’s plots. Bar length indicates duration of treatment. Red circle indicates time when response was observed. Diamond indicates time when progression was noted. Arrowheads indicate ongoing study treatment.

**Figure 3:**
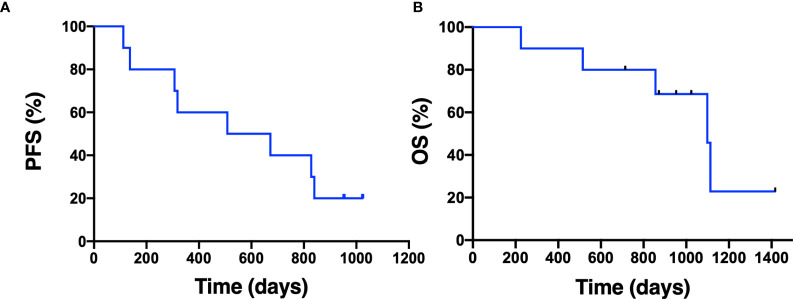
Progression-free survival and overall survival. The Kaplan-Meier estimate for progression-free survival **(A)** and overall survival **(B)** is shown. Censored data are indicated by tick marks.

Median duration of follow-up was 31.3 months. Eight patients had a confirmed partial response (PR), one patient had an unconfirmed response (uPR), and one patient had stable disease as best response (**Figure 1**). The patient with uPR was taken off study because of the need for anticoagulation for deep vein thrombosis (DVT) and the concern about increased bleeding risk due to interaction between dasatinib and the anticoagulant. Median duration of response was 18.6 months (95% CI 8.4 months – not reached). One (10%) of 10 patients experienced intrinsic resistance defined by disease progression or stable disease lasting 4 months or less as best response (**Figure 2**). However, the patient (patient ID 9) likely had two metachronous primary cancers because one of the two target lesions that progressed was found to not harbor an EGFR mutation (the target lesion harboring the *EGFR* mutation remained stable on treatment). Three patients (patient ID 3, 7, 8) were still receiving the study treatment at the time of data cutoff and they have been on study for 46, 33, 31 months, respectively (**Figure 2**). Median PFS was 19.4 months (95% CI 10.1 months – not reached) and median OS was 36.1 months (95% CI 28.1 months – not reached) (**Figure 3**). Subsequent systemic therapies are listed in **Supplemental Table 3**.

### Pharmacokinetics

Cmax and Tmax of osimertinib during cycle 1 were 222.3 nM and 4 hours, respectively (**Supplemental Figure 1**). The geometric mean osimertinib plasma concentrations sharply increased after initiation of the treatment, followed by a steady state (**Supplemental Figure 2A**). Patient ID 9 was found to have a grade 3 rash 9 days after she started the study treatment (**Supplemental Figure 3**). The patient’s pre-treatment osimertinib level on cycle 2 day 1 was 1430 nM, when the levels of osimertinib ranged from 66.4 – 848 nM in other patients at the same time point. Patient ID 10 had lower osimertinib levels than the other patients, but still achieved a confirmed PR (42% reduction in target lesions).

### Serum CRIPTO

Patient ID 1 with decreasing levels of CRIPTO from over 4000 pg/mL achieved a partial response (**Supplemental Figure 4**). Patient ID 2 who had increasing levels of CRIPTO starting at 20 months was found to have progressive disease 27 months after initiation of study treatment. Pre-treatment CRIPTO levels were not associated with PFS in the Cox regression model (data not shown).

### FDG-PET

**Figure 4:**
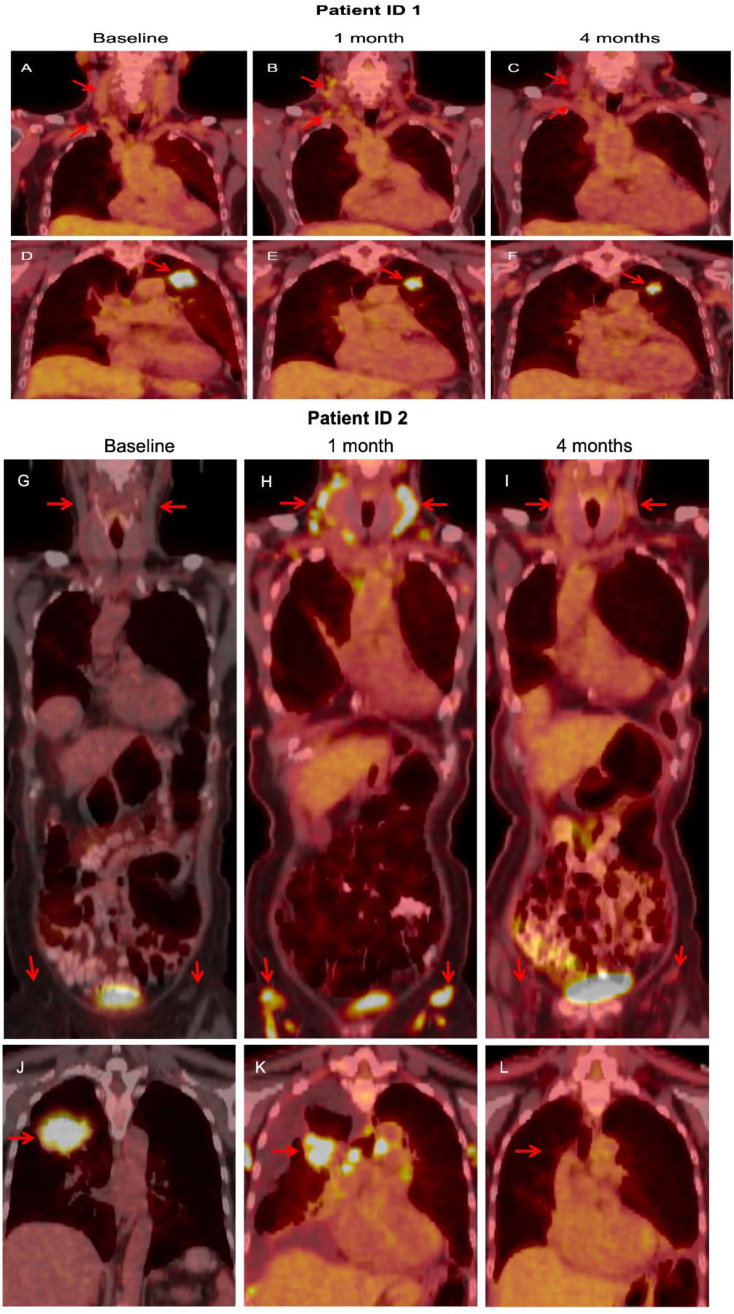
In patient ID 1, FDG-PET obtained after 1 cycle showed diffuse update in cervical lymph nodes **(B)**, compared with baseline **(A)**, which resolved at 4 months **(C)**. The primary lung lesion continued to shrink with study treatment **(D–F)**. In patient ID 2, a similar pattern was noted. FDG-PET obtained after 1 cycle showed diffuse update in cervical and inguinal lymph nodes **(G)**, compared with baseline **(H)**, which resolved at 4 months **(I)**. The primary lung mass responded to study treatment **(J–L)**.

Six (60%) of 10 patients had diffuse FDG uptake in lymph nodes in various locations (**Figure 4**) on the PET scan performed after 28 days. On the PET scan performed after 28 days, primary lung lesions exhibited reduction of size as well as reduced FDG-uptake except for patient ID 9 who had stable disease per RECIST. Follow-up PET showed resolution of the diffuse nodal FDG uptake. There was no difference in PFS between patients with and without reactivation of lymph nodes (data not shown).

## Discussion

Osimertinib has been established as a preferred first-line treatment option for *EGFR*-mutant NSCLC. While effective and well tolerated, clinical utility of osimertinib is limited by intrinsic and acquired resistance. While various mechanisms of resistance to osimertinib have been identified (10, 11), intrinsic resistance to osimertinib has not been well characterized. Studies suggest *MET* amplification, *ERBB2* amplification, and overexpression of AXL as potential mechanisms of intrinsic resistance (12–14). We have shown that CRIPTO through Src activation is implicated in intrinsic resistance to EGFR-TKI therapy (4).

In this phase I trial of osimertinib and the Src inhibitor dasatinib, we demonstrated that it is feasible to combine osimertinib and dasatinib. The combination treatment was associated with mainly grade 1 or 2 adverse events, but chronic adverse events primarily attributable to dasatinib made it challenging for patients to stay on higher dose levels of dasatinib and necessitated multiple dose reductions. Two patients discontinued dasatinib due to dasatinib-related pleural effusion. The lowest dose of dasatinib (50 mg once daily) was generally well tolerated in combination with osimertinib with two patients still receiving treatment on this dose level for 43 and 28 months, respectively. Pleural effusion, which is a known adverse event of dasatinib, occurred in all patients with 90% being grade 1 or 2. Pleural effusions that affected quality of life due to the need for thoracentesis or indwelling catheter occurred in 40% of the patients. Hematologic adverse events were more frequent with the combination when compared with historical data on osimertinib alone from FLAURA. Grade 3 neutropenia occurred in three (30%) patients, but there was no incidence of febrile neutropenia and with dose interruption and reduction of dasatinib, neutropenia improved. The MTD was not determined by protocol-defined DLT criteria due to premature closure of the study; however, given the overall tolerability and toxicity burden at dose levels 1 and 2, the RP2D was chosen as dose level -2 (osimertinib 80 mg once daily plus dasatinib 50 mg once daily). The median PFS and OS and the duration of response associated with osimertinib plus dasatinib were comparable to those observed in FLAURA (2, 3), suggesting that there was no adverse effect of dasatinib on treatment outcomes. However, due to the small size inherent in phase I studies, it is difficult to draw firm conclusions about the efficacy of the combination. The frequent dose reductions and interruptions of dasatinib due to toxicities likely made any potential incremental benefit from the combination to not be discernible. Four (40%) patients had brain metastases at baseline with 2 patients treated with radiotherapy prior to enrollment and 2 patients with untreated brain metastases. None of the patients experienced central nervous system (CNS) progression during study treatment, reflecting the potent intracranial activity of osimertinib (15).

Dasatinib is a multikinase inhibitor that can target several molecules including BCR-ABL, c-KIT, PDGFR-α, and ephrin receptor kinase in addition to Src family kinases (Src, LCK, YES, FYN) (16). Preclinical studies suggest synergistic effects between EGFR-TKI therapy and dasatinib in *EGFR*-mutant NSCLC (4, 17, 18). Single agent activity of dasatinib has been poor in unselected advanced NSCLC and *EGFR*-mutant NSCLC (19–22). Clinical trials combining dasatinib with first- and second-generation EGFR-TKIs have reported no objective response in patients with *EGFR*-mutant NSCLC resistant to prior EGFR-TKI therapy (22, 23) and a low response rate in unselected advanced NSCLC (24). Dasatinib-related toxicities including dyspnea, pleural effusion, fatigue, and gastrointestinal adverse events were frequently encountered in these trials, necessitating dose interruptions and reductions of dasatinib, as in our study. Overall, these results suggest that developing predictive biomarkers of response is essential to the success of the strategy combining Src inhibition with EGFR-targeting therapy. Also, use of more selective Src inhibitors in combination with EGFR-targeting therapy with better tolerability should be considered for future drug development. Saracatinib, a dual kinase inhibitor of Src and Abl, demonstrated a better safety profile in a phase II trial in unselected NSCLC in the second-line setting and beyond. Though the ORR was low, one of the responders had *EGFR*-mutant NSCLC, suggesting potential activity in *EGFR*-mutant NSCLC (25). Unfortunately, the clinical development of saracatinib was discontinued and was not available for our study.

An intriguing finding in our study was that 60% of the patients in the study had diffuse FDG uptake in lymph nodes on PET, which was interpreted as lymphoma or reaction to immunotherapy by radiologists in some cases. Patients remained largely asymptomatic from this phenomenon and the FDG-uptake resolved on follow-up PET. In studies using FDG-PET to assess response to EGFR-TKI treatment, no similar findings were observed (26). Also, in a phase II study of dasatinib in advanced NSCLC where FDG-PET was obtained at baseline and at 6 and 12 weeks, no such phenomenon was documented (20). It has been reported in the literature that dasatinib can rarely cause follicular hyperplasia, leading to increased tracer uptake on PET (27–30). The exact pathogenesis of dasatinib-associated follicular hyperplasia and its clinical implications are unknown. Interestingly, dasatinib has been shown to have an impact on immune cells, resulting in rapid lymphocyte mobilization and activation, which was not seen with other BCR-ABL inhibitors (31). Immune modulating effects of dasatinib may merit further exploration.

Previously, we have demonstrated that soluble CRIPTO was not able to elicit resistance to osimertinib (5). Consistent with this observation, baseline serum CRIPTO level was not associated with PFS in the current study. Of note, we observed increasing serum CRIPTO levels prior to disease progression in some patients. Whether serum CRIPTO can serve as a biomarker for tumor burden in patients with NSCLC deserves further investigation. The phase I portion of the study did not require availability of tissue suitable for evaluation of CRIPTO expression. Due to the lack of available archival tissue samples and premature closure of the trial, we were not able to fully determine the impact of membrane-bound CRIPTO on treatment outcomes.

The Cmax and Tmax of osimertinib after a single dose of osimertinib and dasatinib were similar to those observed in prior pharmacokinetics studies of osimertinib (32, 33). A prior study of pharmacokinetics of osimertinib observed that a steady state was achieved by 15 days of dosing (34). Similarly, we observed that a plateau was achieved by cycle 2 and beyond. It has been shown that pharmacokinetics parameters of erlotinib were not affected by dasatinib (24). Per visual observation, there was no notable difference in osimertinib levels between dasatinib dose level 1 and 2, suggesting no major impact of dasatinib on the level of osimertinib. It was found that the plasma concentration of osimertinib in patient ID 9 was higher than in other patients, which likely explains the degree of rash the patient experienced. The plasma concentrations of AZ13575104 were lower than those of osimertinib, in line with previous studies (34).

## Conclusions

In this study, the combination of osimertinib and dasatinib has shown anti-tumor activity in patients with *EGFR*-mutant NSCLC in the front-line setting, but the treatment was limited by chronic toxicities mainly attributed to dasatinib. In order to improve the safety and tolerability of Src and EGFR co-inhibition, Src inhibitors with a more favorable safety and tolerability profile should be utilized in future studies. Development of predictive biomarkers will be necessary to identify patients who benefit from this therapeutic strategy utilizing Src and EGFR dual inhibition.

## Data Availability Statement

The original contributions presented in the study are included in the article/**Supplementary Material**. Further inquiries can be directed to the corresponding author.

## Ethics Statement

The studies involving human participants were reviewed and approved by Georgetown University. The patients/participants provided their written informed consent to participate in this study. Written informed consent was obtained from the individual(s) for the publication of any potentially identifiable images or data included in this article.

## Author Contributions

Conception or design of the work: CK & GG. Data collection: All authors Data analysis and interpretation: All authors Drafting the article: CK & GG. All authors contributed to the article and approved the submitted version.

## Funding

AstraZeneca provided support in the conduct of the study. The funder was not involved in the study design, collection, analysis, interpretation of data, the writing of this article or the decision to submit it for publication.

## Conflict of Interest

CK reports serving as a consultant or advisory board member for Novartis, Janssen, and PierianDx, and reports research funding (to institution) from AstraZeneca, Bristol-Myers Squibb, Novartis, Genentech, Regeneron, Spectrum, Mirati, Debiopharm, Karyopharm, and Janssen. SL reports serving as a consultant or advisory board member for Amgen, AstraZeneca, Beigene, Blueprint, Bristol-Myers Squibb, Daiichi Sankyo, G1 Therapeutics, Genentech, Guardant Health, Inivata, Janssen, Jazz Pharmaceuticals, Lilly, Merck, PharmaMar, Pfizer, Regeneron and Takeda and reports research funding (to institution) from Alkermes, AstraZeneca, Bayer, Blueprint, Bristol-Myers Squibb, Corvus, Elevation Oncology, Genentech, Lilly, Lycera, Merck, Merus, Pfizer, Rain Therapeutics, RAPT, Spectrum, and Turning Point Therapeutics. VC reports research funding from Bayer. GE reports serving as a consultant for Novartis. DS is currently an employee and shareholder of AstraZeneca Plc. This study received funding from AstraZeneca. The funder had no role in study design, data collection and analysis, decision to publish, or preparation of the manuscript. GG reports serving on advisory boards for Novartis and Daiichi, and research funding from Incyte and Karyopharm.

The remaining authors declare that the research was conducted in the absence of any commercial or financial relationships that could be construed as a potential conflict of interest.

## Publisher’s Note

All claims expressed in this article are solely those of the authors and do not necessarily represent those of their affiliated organizations, or those of the publisher, the editors and the reviewers. Any product that may be evaluated in this article, or claim that may be made by its manufacturer, is not guaranteed or endorsed by the publisher.

## References

[1] Kim C Liu SV. First-Line EGFR TKI Therapy in Non-Small-Cell Lung Cancer: Looking Back Before Leaping Forward. Ann Oncol (2019) 30(12):1852–5. 10.1093/annonc/mdz415 31613313

[2] Soria JC Ohe Y Vansteenkiste J Reungwetwattana T Chewaskulyong B Lee KH. Osimertinib in Untreated EGFR-Mutated Advanced Non-Small-Cell Lung Cancer. N Engl J Med (2018) 378(2):113–25. 10.1056/NEJMoa1713137 29151359

[3] Ramalingam SS Soria JC. Osimertinib in EGFR-Mutated Advanced NSCLC. Reply. N Engl J Med (2020) 382(19):1864–5. 10.1056/NEJMoa1913662 32374973

[4] Park KS Raffeld M Moon YW Xi L Bianco C Pham T. CRIPTO1 Expression in EGFR-Mutant NSCLC Elicits Intrinsic EGFR-Inhibitor Resistance. J Clin Invest (2014) 124(7):3003–15. 10.1172/JCI73048 PMC4071378 24911146

[5] Chen V Iwama E Kim IK Giaccone G. Serum CRIPTO Does Not Confer Drug Resistance Against Osimertinib But is an Indicator of Tumor Burden in non-Small Cell Lung Cancer. Lung Cancer (2020) 145:48–57. 10.1016/j.lungcan.2020.04.032 32408132

[6] Simon R Rubinstein L Arbuck SG Christian MC Freidlin B Collins J. Accelerated Titration Designs for Phase I Clinical Trials in Oncology. J Natl Cancer Inst (1997) 89(15):1138–47. 10.1093/jnci/89.15.1138 9262252

[7] Mitchell R Bailey C Ewles M Swan G Turpin P. Determination of Osimertinib in Human Plasma, Urine and Cerebrospinal Fluid. Bioanalysis (2019) 11(10):987–1001. 10.4155/bio-2018-0262 31218898

[8] Eisenhauer EA Therasse P Bogaerts J Schwartz LH Sargent D Ford R. New Response Evaluation Criteria in Solid Tumours: Revised RECIST Guideline (Version 1.1). Eur J Cancer (2009) 45(2):228–47. 10.1016/j.ejca.2008.10.026 19097774

[9] Tan MT Xiong X. A Flexible Multi-Stage Design for Phase II Oncology Trials. Pharm Stat (2011) 10(4):369–73. 10.1002/pst.478 22328328

[10] Leonetti A Sharma S Minari R Perego P Giovannetti E Tiseo M. Resistance Mechanisms to Osimertinib in EGFR-Mutated non-Small Cell Lung Cancer. Br J Cancer (2019) 121(9):725–37. 10.1038/s41416-019-0573-8 PMC6889286 31564718

[11] Roper N Brown AL Wei JS Pack S Trindade C Kim C. Clonal Evolution and Heterogeneity of Osimertinib Acquired Resistance Mechanisms in EGFR Mutant Lung Cancer. Cell Rep Med (2020) 1(1):100007. 10.1016/j.xcrm.2020.100007 32483558 PMC7263628

[12] Ortiz-Cuaran S Scheffler M Plenker D Dahmen L Scheel AH Fernandez-Cuesta L. Heterogeneous Mechanisms of Primary and Acquired Resistance to Third-Generation EGFR Inhibitors. Clin Cancer Res (2016) 22(19):4837–47. 10.1158/1078-0432.CCR-15-1915 27252416

[13] Taniguchi H Yamada T Wang R Tanimura K Adachi Y Nishiyama A. AXL Confers Intrinsic Resistance to Osimertinib and Advances the Emergence of Tolerant Cells. Nat Commun (2019) 10(1):259. 10.1038/s41467-018-08074-0 30651547 PMC6335418

[14] Xu C Wang W Zhu Y Yu Z Zhang H Wang H. Potential Resistance Mechanisms Using Next Generation Sequencing From Chinese EGFR T790M+ non-Small Cell Lung Cancer Patients With Primary Resistance to Osimertinib: A Multicenter Study. Ann Oncol (2019) 30:ii48. 10.1093/annonc/mdz063.012

[15] Reungwetwattana T Nakagawa K Cho BC Cobo M Cho EK Bertolini A. CNS Response to Osimertinib Versus Standard Epidermal Growth Factor Receptor Tyrosine Kinase Inhibitors in Patients With Untreated EGFR-Mutated Advanced Non-Small-Cell Lung Cancer. J Clin Oncol (2018) 36(33):3290–7. 10.1200/JCO.2018.78.3118 30153097

[16] Lindauer M Hochhaus A. Dasatinib. Recent Results Cancer Res (2018) 212:29–68. 10.1007/978-3-319-91439-8_2 30069624

[17] Wang M Yuang-Chi Chang A. Molecular Mechanism of Action and Potential Biomarkers of Growth Inhibition of Synergistic Combination of Afatinib and Dasatinib Against Gefitinib-Resistant non-Small Cell Lung Cancer Cells. Oncotarget (2018) 9(23):16533–46. 10.18632/oncotarget.24814 PMC5893260 29662665

[18] Watanabe S Yoshida T Kawakami H Takegawa N Tanizaki J Hayashi H. T790M-Selective EGFR-TKI Combined With Dasatinib as an Optimal Strategy for Overcoming EGFR-TKI Resistance in T790M-Positive Non-Small Cell Lung Cancer. Mol Cancer Ther (2017) 16(11):2563–71. 10.1158/1535-7163.MCT-17-0351 28839001

[19] Brunner AM Costa DB Heist RS Garcia E Lindeman NI Sholl LM. Treatment-Related Toxicities in a Phase II Trial of Dasatinib in Patients With Squamous Cell Carcinoma of the Lung. J Thorac Oncol (2013) 8(11):1434–7. 10.1097/JTO.0b013e3182a47162 PMC3801424 24128713

[20] Johnson FM Bekele BN Feng L Wistuba I Tang XM Tran HT. Phase II Study of Dasatinib in Patients With Advanced Non-Small-Cell Lung Cancer. J Clin Oncol (2010) 28(30):4609–15. 10.1200/JCO.2010.30.5474 PMC2974341 20855820

[21] Kelley MJ Jha G Shoemaker D Herndon JE 2nd Gu L Barry WT. Phase II Study of Dasatinib in Previously Treated Patients With Advanced Non-Small Cell Lung Cancer. Cancer Invest (2017) 35(1):32–5. 10.1080/07357907.2016.1253710 27911119

[22] Johnson ML Riely GJ Rizvi NA Azzoli CG Kris MG Sima CS. Phase II Trial of Dasatinib for Patients With Acquired Resistance to Treatment With the Epidermal Growth Factor Receptor Tyrosine Kinase Inhibitors Erlotinib or Gefitinib. J Thorac Oncol (2011) 6(6):1128–31. 10.1097/JTO.0b013e3182161508 PMC3230574 21623279

[23] Creelan BC Gray JE Tanvetyanon T Chiappori AA Yoshida T Schell MJ. Phase 1 Trial of Dasatinib Combined With Afatinib for Epidermal Growth Factor Receptor- (EGFR-) Mutated Lung Cancer With Acquired Tyrosine Kinase Inhibitor (TKI) Resistance. Br J Cancer (2019) 120(8):791–6. 10.1038/s41416-019-0428-3 PMC6474279 30880334

[24] Haura EB Tanvetyanon T Chiappori A Williams C Simon G Antonia S. Phase I/II Study of the Src Inhibitor Dasatinib in Combination With Erlotinib in Advanced Non-Small-Cell Lung Cancer. J Clin Oncol (2010) 28(8):1387–94. 10.1200/JCO.2009.25.4029 PMC3040065 20142592

[25] Laurie SA Goss GD Shepherd FA Reaume MN Nicholas G Philip L. A Phase II Trial of Saracatinib, an Inhibitor of Src Kinases, in Previously-Treated Advanced Non-Small-Cell Lung Cancer: The Princess Margaret Hospital Phase II Consortium. Clin Lung Cancer (2014) 15(1):52–7. 10.1016/j.cllc.2013.08.001 24169259

[26] van Gool MH Aukema TS Hartemink KJ Valdes Olmos RA van Tinteren H Klomp HM. FDG-PET/CT Response Evaluation During EGFR-TKI Treatment in Patients With NSCLC. World J Radiol (2014) 6(7):392–8. 10.4329/wjr.v6.i7.392 PMC4109090 25071879

[27] Pilalas D Koletsa T Arsos G Panselinas G Exadaktylou P Polychronopoulos G. Dasatinib Associated Lymphadenopathy in a Chronic Myeloid Leukemia Patient: A Case Report. Medicine (2020) 99(45):e22791. 10.1097/MD.0000000000022791 33157925 PMC7647569

[28] Ozawa MG Ewalt MD Gratzinger D. Dasatinib-Related Follicular Hyperplasia: An Underrecognized Entity With Characteristic Morphology. Am J Surg Pathol (2015) 39(10):1363–9. 10.1097/PAS.0000000000000488 26360368

[29] Bouquet E Jourdain A Machet MC Beau-Salinas F Jonville-Bera AP. Dasatinib-Associated Follicular Lymphoid Hyperplasia: First Pediatric Case Report and Literature Review. Pediatr Blood Cancer (2017) 64(11):e26597. 10.1002/pbc.26597 28439970

[30] Iurlo A Bucelli C Cattaneo D Orofino N Giannotta JA Zappa M. Reactive Follicular Hyperplasia on Dasatinib Treatment for Chronic Myeloid Leukemia. Ann Hematol (2017) 96(11):1953–4. 10.1007/s00277-017-3105-8 28823087

[31] Mustjoki S Auvinen K Kreutzman A Rousselot P Hernesniemi S Melo T. Rapid Mobilization of Cytotoxic Lymphocytes Induced by Dasatinib Therapy. Leukemia (2013) 27(4):914–24. 10.1038/leu.2012.348 23192016

[32] Zhao H Cao J Chang J Zhang Z Yang L Wang J. Pharmacokinetics of Osimertinib in Chinese Patients With Advanced NSCLC: A Phase 1 Study. J Clin Pharmacol (2018) 58(4):504–13. 10.1002/jcph.1042 29239002

[33] Brown K Comisar C Witjes H Maringwa J de Greef R Vishwanathan K. Population Pharmacokinetics and Exposure-Response of Osimertinib in Patients With non-Small Cell Lung Cancer. Br J Clin Pharmacol (2017) 83(6):1216–26. 10.1111/bcp.13223 PMC5427226 28009438

[34] Planchard D Brown KH Kim DW Kim SW Ohe Y Felip E. Osimertinib Western and Asian Clinical Pharmacokinetics in Patients and Healthy Volunteers: Implications for Formulation, Dose, and Dosing Frequency in Pivotal Clinical Studies. Cancer Chemother Pharmacol (2016) 77(4):767–76. 10.1007/s00280-016-2992-z 26902828

