# Supplementary material for: A Phase I Trial of Dasatinib and Osimertinib in TKI Naïve Patients With Advanced EGFR-Mutant Non-Small-Cell Lung Cancer
Source: Front Oncol. 2021 Sep 8;11:728155. doi: 10.3389/fonc.2021.728155 (PMC8457399; doi:10.3389/fonc.2021.728155)

Supplemental Figure legends

Supplemental Figure 1. Mean osimertinib concentration-time profile following the administration of osimertinib and dasatinib on cycle 1 day 1.

Supplemental Figure 2. Longitudinal mean concentration-time profile of osimertinib (A) and one of its metabolites AZ13575104 (B) during study treatment.

Supplemental Figure 3. Longitudinal osimertinib-time profile of individual patients.

Supplemental Figure 4. Serial CRIPTO levels in serum.

Supplemental Figure 1

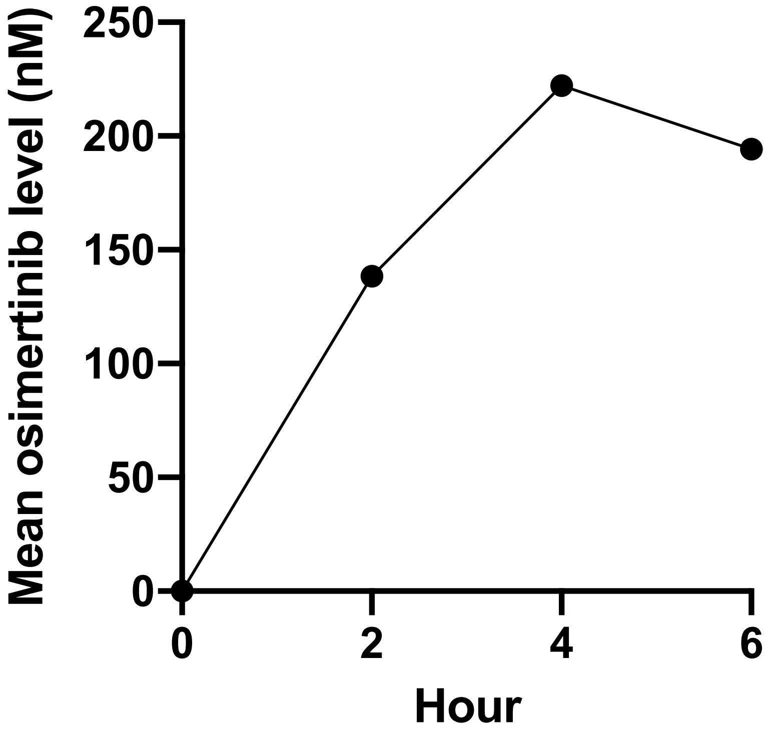

Supplemental Figure 2

A

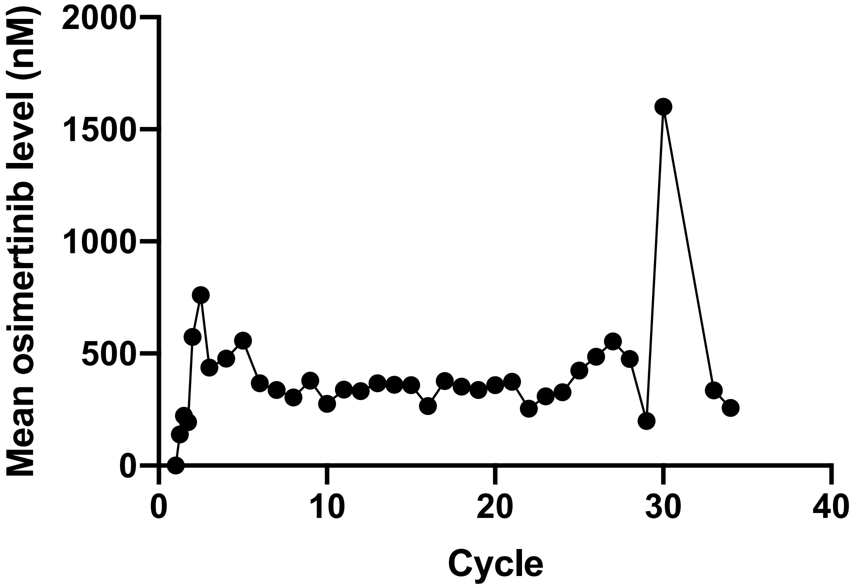

B

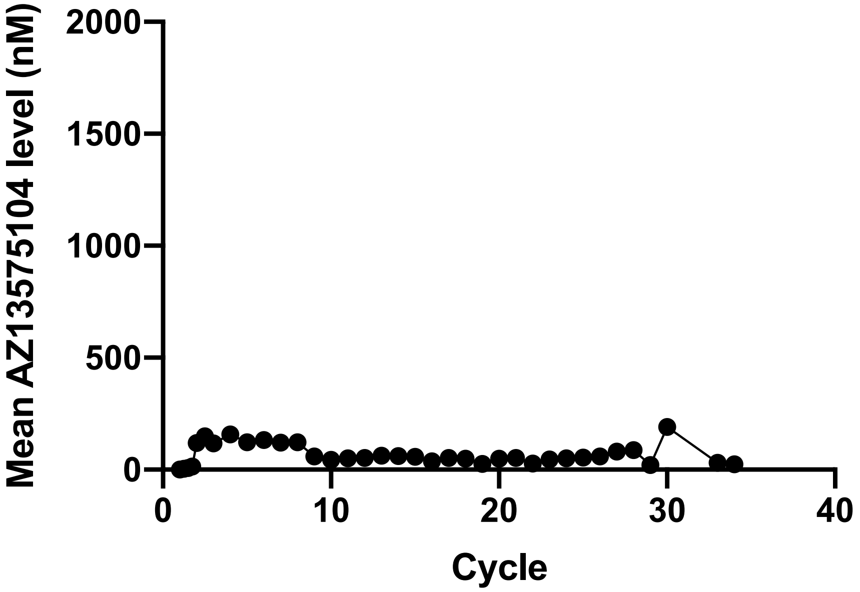

Supplemental Figure 3

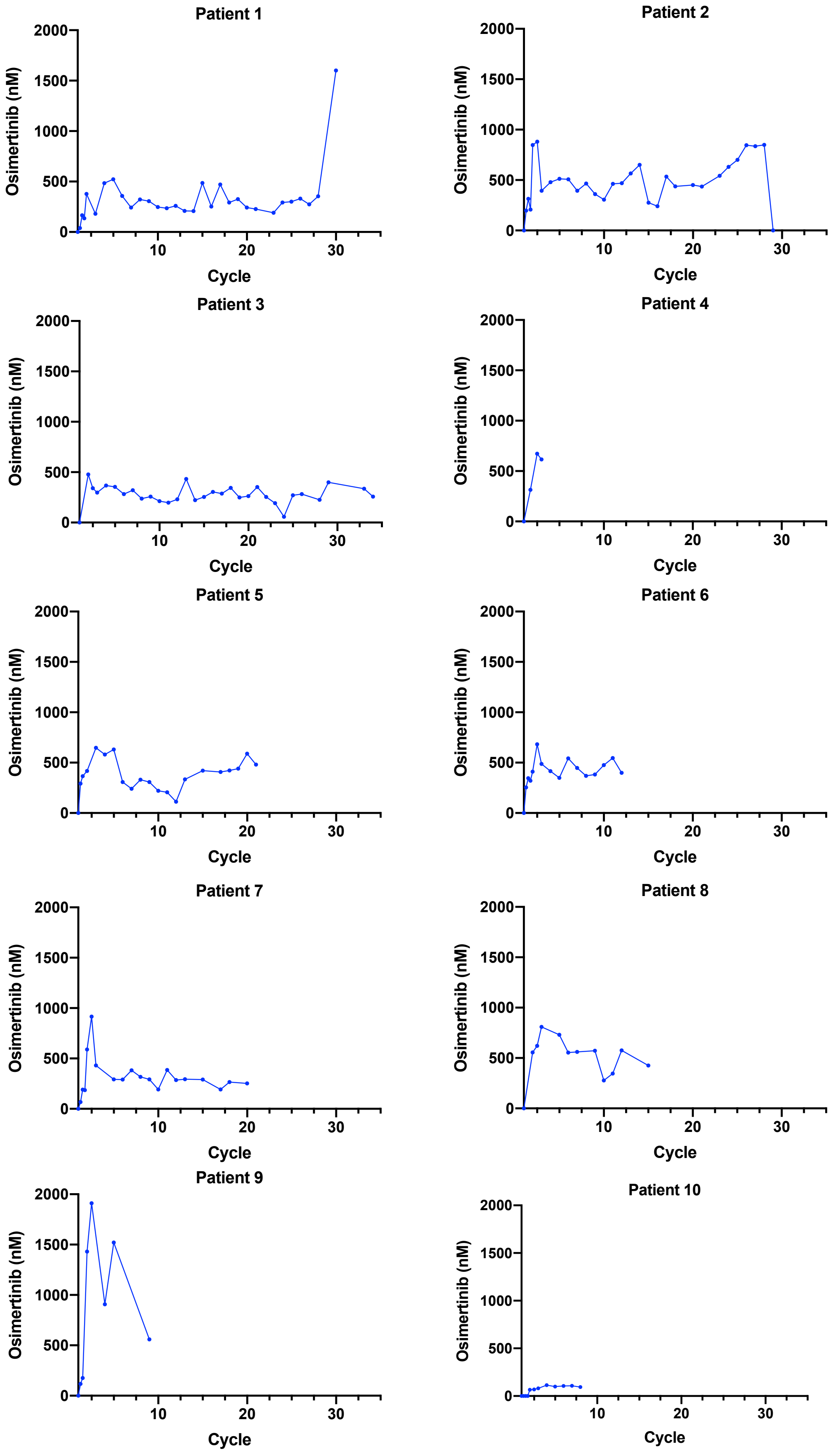

Supplemental Figure 4

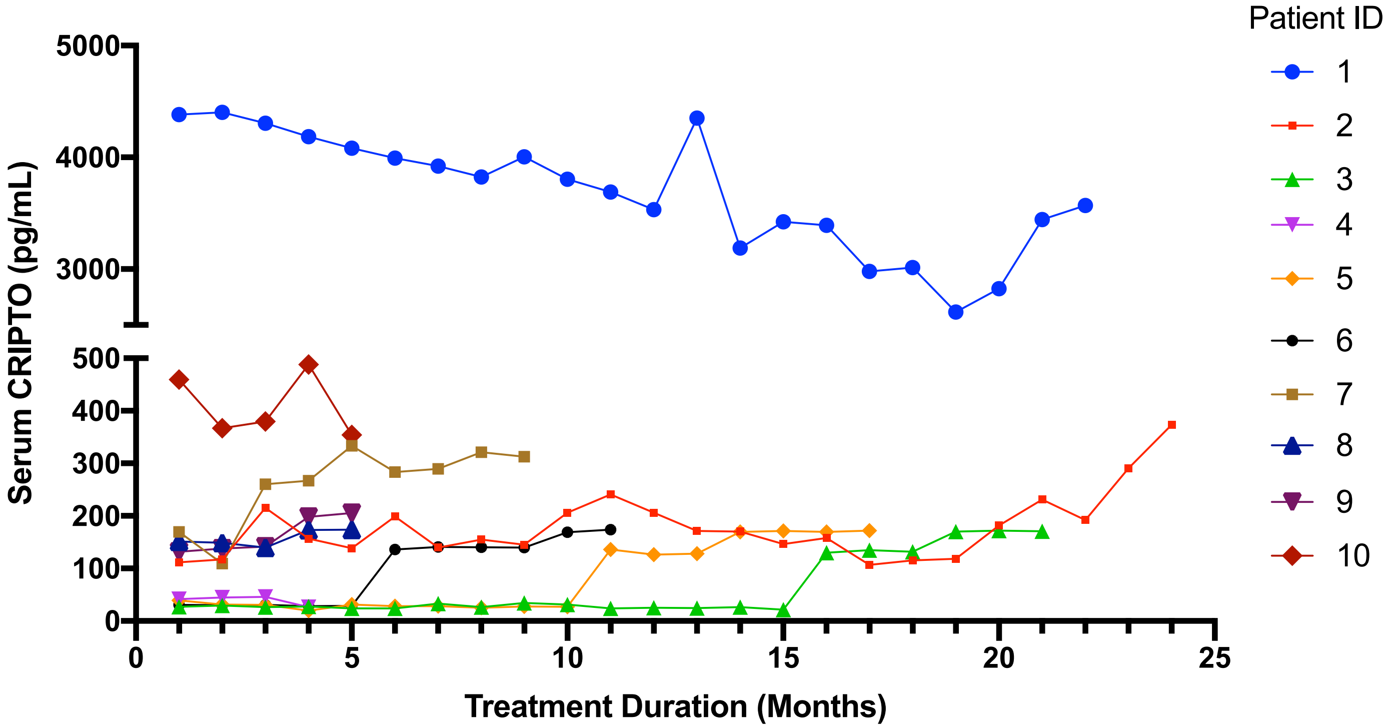

Supplement: Supplementary file 1 [file DataSheet_1.pdf]
