# Supplementary material for: A Phase I Trial of Dasatinib and Osimertinib in TKI Naïve Patients With Advanced EGFR-Mutant Non-Small-Cell Lung Cancer
Source: Front Oncol. 2021 Sep 8;11:728155. doi: 10.3389/fonc.2021.728155 (PMC8457399; doi:10.3389/fonc.2021.728155)
Supplement: Supplementary file 2 [file DataSheet_2.pdf]

Supplemental Table 1. Days on each dose level of dasatinib (number of days off dasatinib due to dose interruption secondary to adverse events in parenthesis)

Patient ID	Dose level	Days on 70 mg twice daily (dose level 2)	Days on 50 mg twice daily (dose level 1)	Days on 70 mg once daily (dose level -1)	Days on 50 mg once daily (dose level -2)
1	1		86 (8)	46 (20)	687 (7)
2	1		279 (0)	65 (11)	462 (49)
3	1		84 (0)	28 (0)	1305 (43)*
4	2	81 (0)			
5	2	70 (9)	28 (3)	21 (0)	
6	2	19 (12)	15 (5)	21 (8)	245 (29)
7	1		5 (3)	52 (14)	697 (82)
8	1		63 (28)	26 (6)	864 (59†)*
9	1		14 (5)	28 (4)	70 (0)
10	-1			112 (21)	221 (0)

* Ongoing treatment

† Patient was additionally off dasatinib between 3/9/2020 - 5/14/2020 due to the worsening COVID-19 pandemic situation and the concern about neutropenia on dasatinib. Patient has a history of AML now in remission.

Supplemental Table 2. Reasons for dose reduction and discontinuation.

Patient ID	Dose level	Reason for dose reduction from dose level 2 to dose level 1	Reason for dose reduction from dose level 1 to dose level -1	Reason for dose reduction from dose level -1 to dose level -2	Reason for dasatinib discontinuation
1	1		Symptomatic atrial fibrillation	Symptomatic atrial fibrillation	Need for anticoagulation for deep vein thrombosis and drug interaction between the anticoagulant and dasatinib
2	1		Pleural effusion	Pleural effusion, fatigue	Pneumonitis
3	1		Fatigue	Fatigue	
4	2				Need for anticoagulation for deep vein thrombosis and drug interaction between the anticoagulant and dasatinib
5	2	Pleural effusion	Pleural effusion		Pleural effusion
6	2	Nausea, fever, fluid retention	Nausea, fluid retention	Dyspnea, chest heaviness	Pleural effusion
7	1		Headaches, myalgia	Pleural effusion	
8	1		QTc prolongation	Neutropenia	
9	1		Rash	Creatinine elevation	Disease progression
10	-1			Nausea, anorexia	Disease progression

Supplemental Table 3. Subsequent therapies used in patients who were taken off the study

Patient ID	Subsequent treatment
1	Osimertinib/necitumumab
2	None
4	Erlotinib<br>Carboplatin/pemetrexed
5	Osimertinib/necitumumab<br>Carboplatin/pemetrexed<br>Osimertinib/alectinib
6	Osimertinib/MP0250<br>Carboplatin/pemetrexed/osimertinib
9	RT to the oligoprogressive lesion, followed by gefitinib<br>Carboplatin/pemetrexed/pembrolizumab
10	Osimertinib/necitumumab<br>Carboplatin/pemetrexed

Supplemental Table 4. Geometric means of osimertinib and AZ13575104 per cycle

Time point	Osimertinib	AZ13575104
Cycle 1 (pre-dose)	0.0	0.0
Cycle 1 (2 hour post)	138.4	3.6
Cycle 1 (4 hour post)	222.3	7.0
Cycle 1 (6 hour post)	166.4	15.0
Cycle 2 (pre-dose)	574.8	118.9
Cycle 2 (4 hour post)	761.3	149.3
Cycle 3	563.5	117.8
Cycle 4	478.0	158.1
Cycle 5	716.0	121.9
Cycle 6	420.9	133.2
Cycle 7	384.9	121.1
Cycle 8	305.0	122.5
Cycle 9	433.9	59.6
Cycle 10	275.4	45.0
Cycle 11	339.6	50.2
Cycle 12	333.1	53.0
Cycle 13	262.1	63.2
Cycle 14	154.4	60.5
Cycle 15	307.3	56.7
Cycle 16	113.7	38.1
Cycle 17	270.0	51.7
Cycle 18	251.4	48.7
Cycle 19	145.0	25.1
Cycle 20	256.6	48.7
Cycle 21	213.9	52.2

Cycle 22	36.3	28.1
Cycle 23	132.3	45.8
Cycle 24	140.0	51.1
Cycle 25	181.7	53.8
Cycle 26	208.1	59.3
Cycle 27	158.4	81.0
Cycle 28	204.0	86.9
Cycle 29	57.0	21.1
Cycle 30	228.6	190.0
Cycle 31*	-	-
Cycle 32*	-	-
Cycle 33	48.0	30.0
Cycle 34	257.0	23.6

* Missing samples for cycle 31 and 32
